# Supplementary figures and images for: Wolbachia co-infection in a hybrid zone: discovery of horizontal gene transfers from two Wolbachia supergroups into an animal genome
Source: PeerJ. 2015 Dec 7;3:e1479. doi: 10.7717/peerj.1479 (PMC4675112; doi:10.7717/peerj.1479)

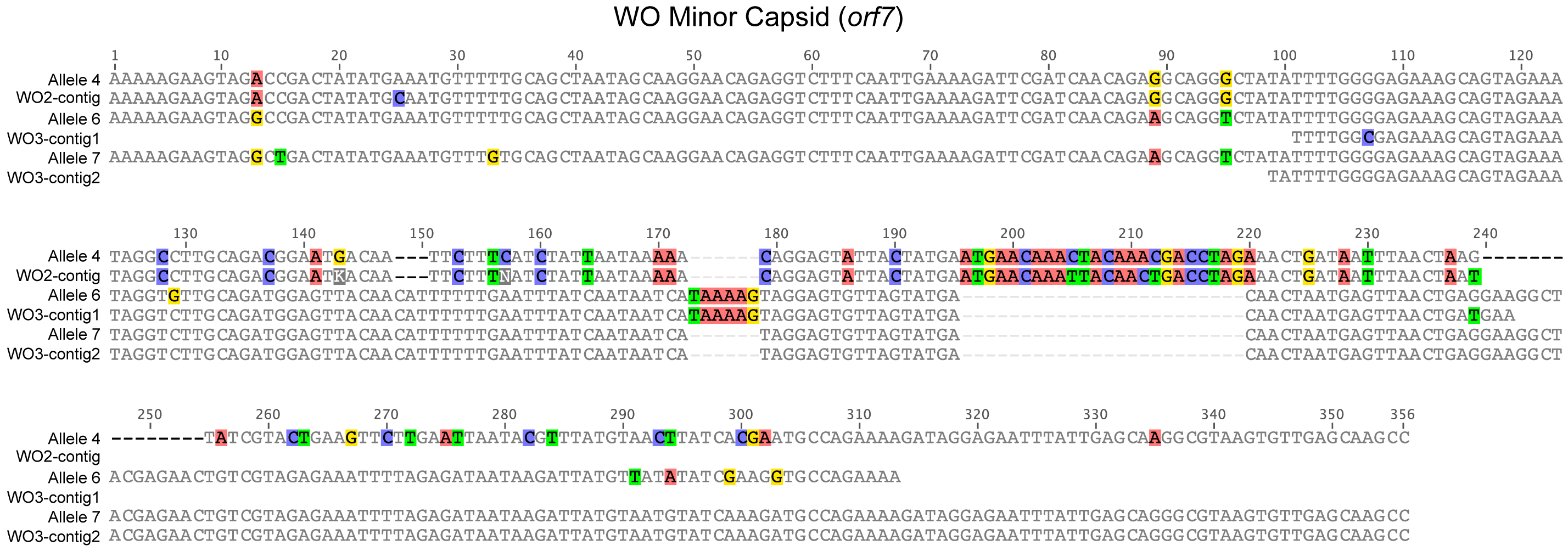

Supplement: Figure S1 — Contigs are grouped with their most similar WO allele identified through Sanger sequencing. Nucleotides are counted from start of the sequence alignment, not from the transcription start site of the gene. [file peerj-03-1479-s001.png]

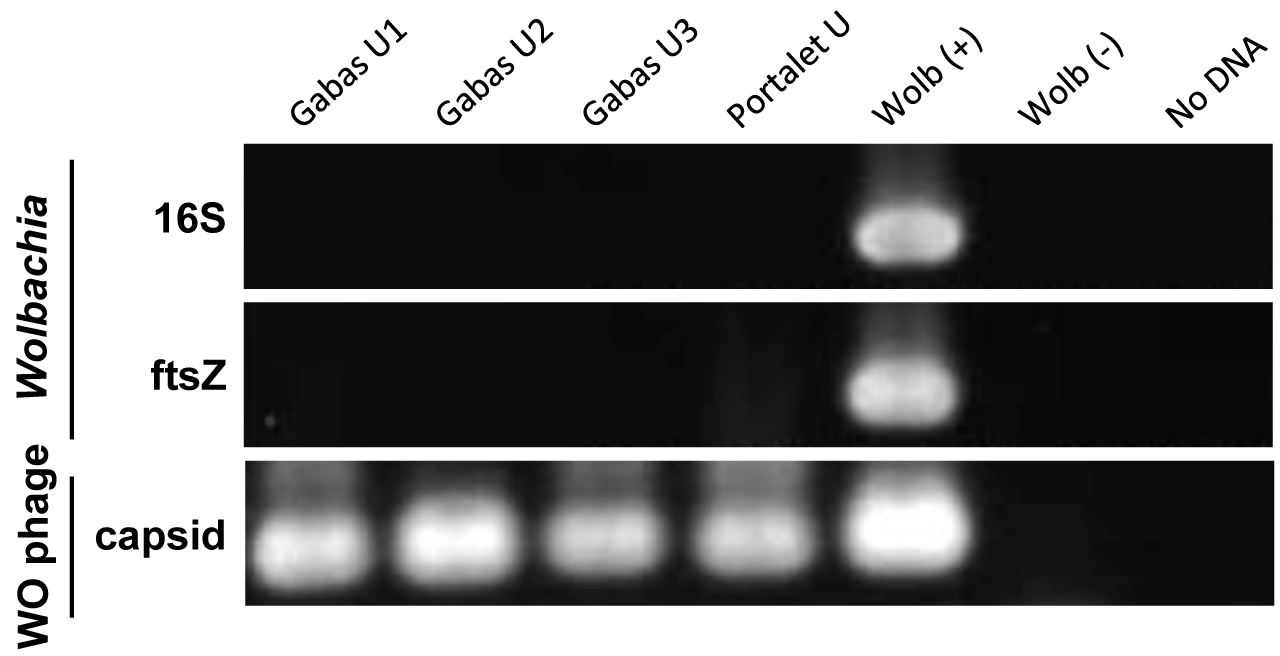

Supplement: Figure S2 — Wolbachia genes (16S rRNA and ftsZ) and the phage WO orf7 gene were amplified from Gabas uninfected grasshoppers used for high-throughput sequencing (Gabas U1–U3) and a Portalet uninfected grasshopper for comparison (Portalet U). Wolb (+) and (−) controls are from Wolbachia-infected and tetracycline-cured lines of Nasonia giraulti, respectively. No DNA, no template added to PCR. [file peerj-03-1479-s002.png]
