# Supplementary material for: Wolbachia co-infection in a hybrid zone: discovery of horizontal gene transfers from two Wolbachia supergroups into an animal genome
Source: PeerJ. 2015 Dec 7;3:e1479. doi: 10.7717/peerj.1479 (PMC4675112; doi:10.7717/peerj.1479)
Supplement: Table S1 — Numbers represent individual grasshoppers. Cpp, Chorthippus parallelus parallelus; Cpe, Chorthippus parallelus erythropus. [file peerj-03-1479-s003.docx]

**Table S1: Sample sizes of PCR screen for WO minor capsid gene**

| Population | Subspecies | Co-infected | F-infected | B-infected | Uninfected |
| --- | --- | --- | --- | --- | --- |
| Portalet | Hybrid | 5 | 4 | 2 | 6 |
| Gabas | Cpp | 2 | 3 | 3 | 0 |
| Navafria | Cpe | 3 | 3 | 3 | 2 |
| Slovenia | Cpp | 0 | 7 | 0 | 0 |

Numbers represent individual grasshoppers. Cpp = *Chorthippus parallelus parallelus*; Cpe = *Chorthippus parallelus erythropus*
