# Supplementary material for: Wolbachia co-infection in a hybrid zone: discovery of horizontal gene transfers from two Wolbachia supergroups into an animal genome
Source: PeerJ. 2015 Dec 7;3:e1479. doi: 10.7717/peerj.1479 (PMC4675112; doi:10.7717/peerj.1479)
Supplement: Table S2 [file peerj-03-1479-s004.docx]

| Individual | Infection Status | Allele 1 | Allele 2 | Allele 3 | Allele 4 | Allele 5 | Allele 7 | Total |
| --- | --- | --- | --- | --- | --- | --- | --- | --- |
| 604FB | F and B | 2 | 0 | 1 | 6 | 5 | 1 | 15 |
| 607F | F only | 0 | 1 | 0 | 5 | 11 | 0 | 17 |
| 603B | B only | 0 | 0 | 1 | 3 | 1 | 3 | 8 |
| 641U | Uninfected | 0 | 0 | 0 | 12 | 4 | 0 | 16 |

**Table S2: Number of sequences obtained through cloning and Sanger sequencing of WO minor capsid gene for individual grasshoppers**
