# Supplementary material for: Wolbachia co-infection in a hybrid zone: discovery of horizontal gene transfers from two Wolbachia supergroups into an animal genome
Source: PeerJ. 2015 Dec 7;3:e1479. doi: 10.7717/peerj.1479 (PMC4675112; doi:10.7717/peerj.1479)
Supplement: Table S3 — All accession numbers are from GenBank unless bolded, which are from the EMBL-EBI database. “No annotation” means that the phage genome is sequenced but not annotated. “N/A” means that the phage genome or phage gene has not been sequenced. [file peerj-03-1479-s005.docx]

**Table S3: Locus tags for WO minor capsid variants used for *orf7* phylogeny**

| WO Haplotype | Minor capsid locus tag | *Wolbachia* strain | NCBI Accession # |
| --- | --- | --- | --- |
| WORiA | WRi_012630 | *w*Ri | CP001391 |
| WORiB-1 | WRi_005560 | *w*Ri | CP001391 |
| WORiB-2 | WRi_010220 | *w*Ri | CP001391 |
| WORiC | WRi_007170 | *w*Ri | CP001391 |
| WOSol | So0014 | *w*Sol | KC955252 |
| WOMelA | WD0271 | *w*Mel | AE017196 |
| WOMelB | WD0602 | *w*Mel | AE017196 |
| WOAu1 | WPWAU_0301 | *w*Au | LK055284 |
| WOAu2 | WPWAU_0654 | *w*Au | LK055284 |
| WOHa1 | wHa_02460 | *w*Ha | CP003884 |
| WOHa2 | wHa_03530 | *w*Ha | CP003884 |
| WOPip1 | WP0252 | *w*Pip (Pel) | AM999887 |
| WOPip2 | WP0311 | *w*Pip (Pel) | AM999887 |
| WOPip3 | WP0326 | *w*Pip (Pel) | AM999887 |
| WOPip4 | WP0426 | *w*Pip (Pel) | AM999887 |
| WOPip5 | WP1303 | *w*Pip (Pel) | AM999887 |
| WOCauB1 | WOCauB1_gp3 | *w*CauB | AB161975 |
| WOCauB2 | WOCauB2_B2gp17 | *w*CauB | AB478515 |
| WOCauB3 | WOCauB3_B3gp18 | *w*CauB | AB478516 |
| WOVitA1 | ADW80142 | *w*VitA | HQ906662 |
| WOVitA2 | No annotation | *w*VitA | HQ906663 |
| WOVitA4 | No annotation | *w*VitA | HQ906664 |
| WOVitB | ADW80201 | *w*VitB | HQ906665 |
| WO-WVulC3-4 | HM452368 | *w*Vul | N/A |
| WO-WVulC6 | HM452370 | *w*Vul | N/A |
| WOTai | wTai_orf7 | *w*Tai | AB036665 |
| WONo1 | wNo_01210 | *w*No | CP003883 |
| WOBol1 | wBol1_1361 | *w*Bol | **CAOH00000000** |
| Orf7 (allele 1) |  |  | KR081343 |
| Cpar-WO1 (allele 2) |  |  | KR081342 |
| Cpar-WO2 (allele 3) |  |  | KR081345 |
| Cpar-WO2 (allele 4) |  |  | KR081346 |
| Cpar-WO2 (allele 5) |  |  | KR081347 |
| Cpar-WO3 (allele 6) |  |  | KT599860 |
| Cpar-WO3 (allele 7) |  |  | KR081344 |
| Cpar-WO3 (allele 8) |  |  | KT599861 |

All accession numbers are from GenBank unless bolded, which are from the EMBL-EBI database. “No annotation” means that the phage genome is sequenced but not annotated. “N/A” means that the phage genome or phage gene has not been sequenced.
