# Supplementary material for: Wolbachia co-infection in a hybrid zone: discovery of horizontal gene transfers from two Wolbachia supergroups into an animal genome
Source: PeerJ. 2015 Dec 7;3:e1479. doi: 10.7717/peerj.1479 (PMC4675112; doi:10.7717/peerj.1479)
Supplement: Table S4 [file peerj-03-1479-s006.docx]

| *Wolb.* genome | NCBI Reference # | *Wolb.* supergroup | # of mapped reads | # of total contigs | Length of longest contig (bp) | Average length of contigs (bp) | Contig N50 (bp) | Total length of reference covered (bp) |
| --- | --- | --- | --- | --- | --- | --- | --- | --- |
| **90% sequence similarity over 90% read length** | | | | | | | | |
| *w*Pip | AM999887 | B | 10,952 | 1,990 | 4,290 | 206 | 255 | 409,978 |
| *w*Cle | AP013028 | F | 3,472 | 921 | 2,063 | 165.1 | 175 | 152,099 |
| *w*Mel | AE017196 | A | 2,517 | 448 | 1,360 | 166.5 | 186 | 74,612 |
| *w*Bm | AE017321 | D | 70 | 51 | 192 | 93.8 | 96 | 4,786 |
| *w*Oo | HE660029 | C | 20 | 16 | 114 | 89.8 | 93 | 1,437 |
| **65% sequence similarity over 80% read length** | | | | | | | | |
| *w*Pip | AM999887 | B | 19,359 | 3,072 | 4,289 | 169.5 | 242 | 520,749 |
| *w*Cle | AP013028 | F | 7,058 | 1,957 | 2,617 | 118.9 | 136 | 232,684 |
| *w*Mel | AE017196 | A | 6,954 | 1,361 | 1,880 | 106.8 | 136 | 145,396 |
| *w*Bm | AE017321 | D | 4,331 | 793 | 456 | 54.6 | 56 | 43,323 |
| *w*Oo | HE660029 | C | 4,760 | 828 | 171 | 47.1 | 45 | 38,997 |

**Table S4: Statistics for reads mapped to *Wolbachia* genomes from multiple supergroups**
